# Trends in US Internal Medicine Residency and Fellowship Applications During the COVID-19 Pandemic vs Previous Years

**DOI:** 10.1001/jamanetworkopen.2021.8199

**Published:** 2021-04-28

**Authors:** Laura A. Huppert, Lekshmi Santhosh, Jennifer M. Babik

**Affiliations:** 1 Hematology/Oncology Division, Department of Medicine, University of California, San Francisco; 2 Pulmonary and Critical Care Division, Department of Medicine, University of California, San Francisco; 3 Infectious Disease Division, Department of Medicine, University of California San Francisco

## Abstract

This cross-sectional study evaluates the number of applicants and number of applications submitted per applicant to internal medicine residency and subspecialty fellowships for 2021 compared with 5 prior application cycles.

## Introduction

The COVID-19 pandemic has significantly affected medical education,^1^ from disrupting trainee schedules to introducing virtual residency and fellowship interviews.^2^ The effect on application patterns to internal medicine (IM) residency and subspecialty fellowships is unknown. We evaluated the number of applicants and number of applications submitted per applicant to IM residency and subspecialty fellowships for 2021 vs the 5 prior application cycles.

## Methods

In this cross-sectional study, we extracted publicly reported Electronic Residency Application Service (ERAS) application data from 2016 to 2021 for IM residency and 11 subspecialty fellowships.^3^ This report followed the Strengthening the Reporting of Observational Studies in Epidemiology (STROBE) reporting guideline for cross-sectional studies.^4^ The University of California, San Francisco institutional review board does not require approval for research using publicly available deidentified data. Data were analyzed using Prism 9.0 (GraphPad).

## Results

### Number of Applicants

For IM residency, the number of applicants increased every year, from 21 947 applicants in 2016 to 24 509 applicants in 2021 (Figure, A). The annual increase from 2020 to 2021 (from 23 121 to 24 509 applicants; 6.0% increase) was more than twice the rate of annual increase in any prior year studied (range, 0.2%-2.7%) (Figure, B). For IM subspecialty fellowships, the number of applicants increased for all 11 subspecialties between 2016 and 2021 (with year-to-year variation) and for all subspecialties except gastroenterology between 2020 and 2021 (Figure, A). From 2020 to 2021, the number of applicants increased substantially for 10 fellowship programs: allergy/immunology (from 192 to 238 applicants; 24.0% increase), hospice/palliative care medicine (HPM) (from 444 to 535 applicants; 20.5% increase), infectious diseases (from 424 to 496 applicants; 17.0% increase), nephrology (from 446 to 521 applicants; 16.8% increase), geriatric medicine (from 293 to 331 applicants; 13.0% increase), cardiology (from 1587 to 1785 applicants; 12.5% increase), endocrinology (from 436 to 485 applicants; 11.2% increase), hematology/oncology (from 909 to 1010 applicants; 11.1% increase), pulmonary/critical care medicine (from 1294 to 1380 applicants; 6.6% increase), and rheumatology (from 418 to 421 applicants; 0.7% increase) (Figure, B). The annual increase from 2020 to 2021 was higher than all prior years studied for 7 of the 11 fellowships (63.6%; including allergy/immunology, cardiology, endocrinology, geriatric medicine, hematology/oncology, HPM, and nephrology). Only gastroenterology had a decrease in the number of applicants from 2020 to 2021 (from 1047 to 1044 applicants; 0.3% decrease).

**Figure.**
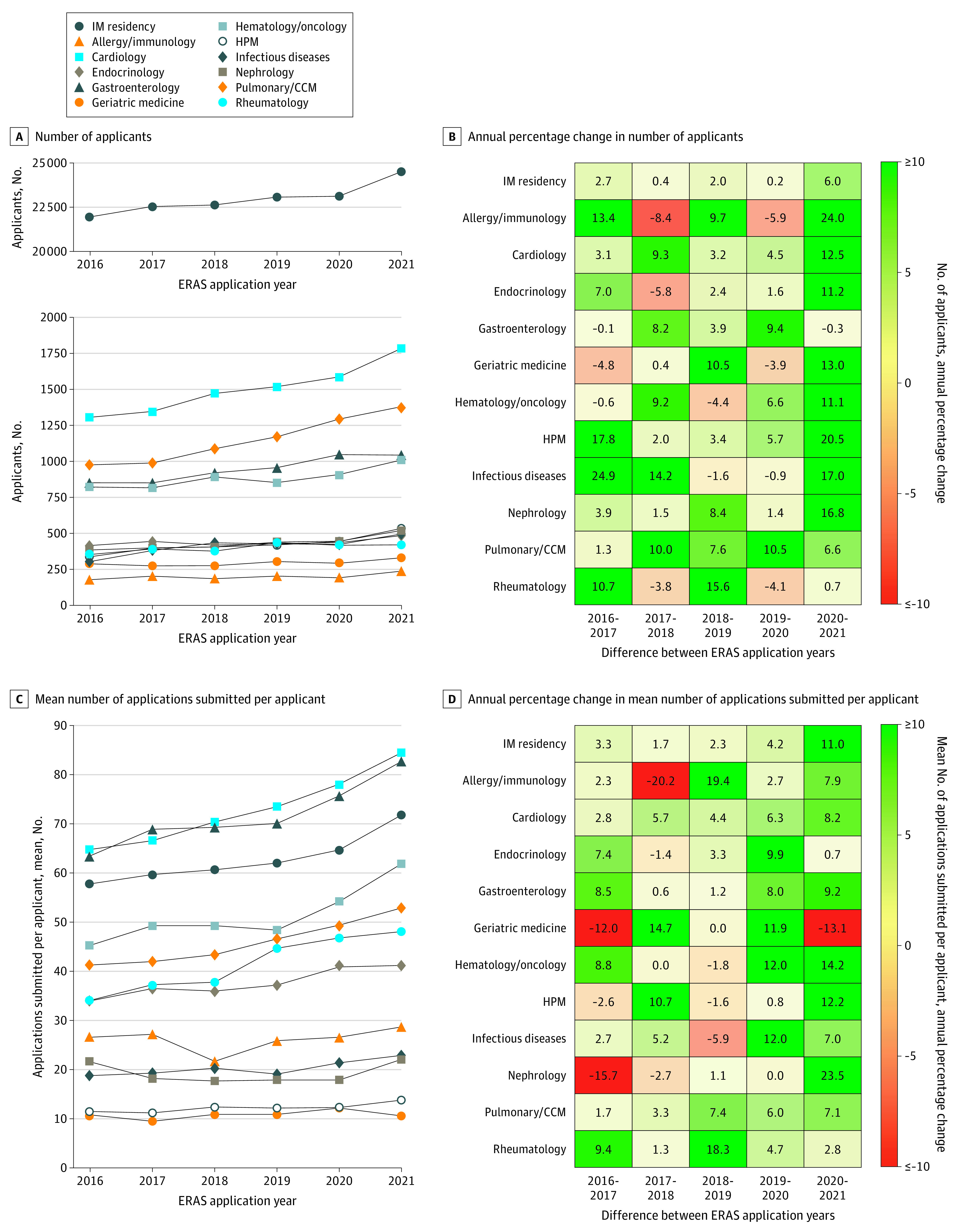
Trends in the Number of Applicants and the Mean Number of Applications Submitted per Applicant for Internal Medicine (IM) Residency and Subspecialty Fellowships During the 2016 to 2021 Electronic Residency Application Service (ERAS) Submission Cycles CCM indicates critical care medicine; and HPM, hospice/palliative care medicine.

### Number of Applications Submitted per Applicant

For IM residency, the mean number of applications submitted per applicant increased annually, from 57.8 applications submitted per applicant in 2016 to 71.8 applications submitted per applicant in 2021 (Figure, C). The annual increase from 2020 to 2021 (7.1 additional applications submitted per applicant; 11.0% increase) was more than twice the rate of annual increase than in any prior year studied (range, 1.7%-4.2%) (Figure, D). For IM subspecialty fellowships, the average number of applications submitted per applicant increased for all subspecialties except geriatric medicine between 2016 and 2021 (with year-to-year variation), including between 2020 and 2021 (Figure, C). For 5 of the 11 subspecialty fellowships (45.5%; including cardiology, gastroenterology, hematology/oncology, HPM, and nephrology), the increase between 2020 and 2021 was higher than all prior years (Figure, D).

## Discussion

In 2021, the number of applicants and the number of applications submitted per applicant for IM residency and subspecialty fellowships increased, with a greater rate of increase for most programs between 2020 and 2021 than in the 5 prior years. We hypothesize that the increase in the number of applicants may be associated with a lower barrier to apply because of decreased time and costs related to virtual interviews or because fellowship training offered more short-term job security in the setting of widespread hiring freezes. Moreover, COVID-19 may have highlighted clinical and/or research opportunities in certain IM fields: substantial increases were noted in 2021 for infectious diseases (17.0%), geriatric medicine (13.0%), HPM (20.5%), and pulmonary/critical care medicine (6.6%). Of note, gastroenterology was the only fellowship with fewer applicants in 2021 (−0.3%), which may have been due to the large increase in applicants in 2020.

The mean number of applications submitted per applicant in 2021 increased for all programs except geriatric medicine (which had experienced a large increase in 2020). Virtual interviews, and the associated time and cost savings for applicants, may have enabled applicants to apply to more programs.^2^ This trend may lead to a more diverse applicant pool^5^; however, there are also potential negative consequences, such as added time for programs to review applications and congestion in the already labor- and time-intensive recruitment process.^6^ As a result, some have proposed caps on the number of applications submitted per applicant,^6^ which may be particularly important if virtual interviews continue as a common practice.

There are limitations to this analysis. We did not evaluate for the factors that are associated with changes in application patterns, and data were not available by gender or race/ethnicity at the time of this analysis. Additional studies are needed to determine the factors associated with these changes, whether they differ by gender or race/ethnicity, the resulting consequences on both applicants and programs, and whether countermeasures, such as application caps, are warranted.
